# The refractive accuracy between topographic keratometry and biometric keratometry for extended depth-of-focus intraocular lens implantation

**DOI:** 10.7150/ijms.99907

**Published:** 2024-11-04

**Authors:** Chia-Yi Lee, Shun-Fa Yang, Hung-Chi Chen, Ie-Bin Lian, Jing-Yang Huang, Chao-Kai Chang

**Affiliations:** 1 Institute of Medicine, Chung Shan Medical University, Taichung, Taiwan.; 2 Nobel Eye Institute, Taipei, Taiwan.; 3 Department of Ophthalmology, Jen-Ai Hospital Dali Branch, Taichung, Taiwan.; 4 Department of Medical Research, Chung Shan Medical University Hospital, Taichung, Taiwan.; 5 Department of Ophthalmology, Chang Gung Memorial Hospital, Linkou, Taiwan.; 6 Center for Tissue Engineering, Chang Gung Memorial Hospital, Linkou, Taiwan.; 7 Department of Medicine, Chang Gung University College of Medicine, Taoyuan, Taiwan.; 8 Institute of Statistical and Information Science, National Changhua University of Education, Taiwan.; 9 Department of Optometry, Yuanpei University of Medical Technology, Hsinchu, Taiwan.

**Keywords:** extended depth-of-focus, topographic, biometric, astigmatism, angle kappa

## Abstract

**Purpose:** To investigate the influence of different keratometry (K) measurements on the postoperative outcomes of cataract surgery with extended depth-of-focus (EDOF) intraocular lens (IOL) implantation.

**Methods:** A retrospective cohort study was conducted, and patients who received cataract surgery and one type of EDOF IOL implantation were included. The patients were then categorized according to K measurements, and 70 and 30 eyes were included in the biometric-K and topographic-K groups, respectively. The primary outcomes were postoperative uncorrected distance visual acuity (UDVA), spherical equivalent (SE) and cylinder power. A generalized linear model was applied to compare the adjusted odds ratios (aORs) and 95% confidence intervals (CIs) of the outcomes between groups.

**Results:** One month after surgery, the UDVA was 0.15 and 0.07 in the biometric-K group and topographic-K group, respectively. Furthermore, the final SEs were -0.42 D and -0.13 D in the biometric-K group and topographic-K group, respectively, and the final cylinder powers were -0.35 D and -0.13 D in the biometric-K group and topographic-K group, respectively. According to the multivariate analysis, the topographic-K group presented a significantly better UDVA (P = 0.044) and significantly lower cylinder power (P = 0.031) than the biometric-K group. Angle kappa was significantly correlated with high postoperative astigmatism in the topographic-K group (P = 0.033), whereas angle kappa, steep K, and corneal cylinder powers were significantly correlated with high postoperative astigmatism in the biometric-K group (all P < 0.05).

**Conclusion:** Topography-based K measurements yielded better refractive outcomes than biometric-based K measurements did.

## Introduction

Cataracts are prevalent ophthalmic disorders that cause blindness in approximately 20 million people worldwide 1. The common symptoms of cataracts include progressively blurry vision and monocular diplopia, which can significantly impair quality of life 2. Currently, the only intervention used to resolve the reduced vision caused by cataracts is cataract surgery 3. In general, postoperative visual acuity is significantly improved after cataract surgery, and multiple types of intraocular lenses (IOLs) can be selected to fulfill the needs of distance and near vision 4, 5.

Despite improvements in postoperative visual acuity, postoperative refractive status and related IOL calculations remain important issues for all cataract surgeries 6-8. The residual refractive error after cataract surgery is associated with a lower patient satisfaction rate 9. Additionally, postoperative residual astigmatism contributes to halo, glare and reduced vision, especially in those with irregular astigmatism 10, 11. In a previous study, the uncorrected distance visual acuity (UDVA) in patients who received multifocal or extended depth-of-focus (EDOF) IOL was reduced with postoperative residual astigmatism 12. Consequently, the accuracy of refractive calculations in these patients cannot be overemphasized.

Several parameters need to be included in the IOL calculation formulas to refine the postoperative refraction 13-15. Among them, the value of corneal refractive power can be obtained via different methods 16. Biometric keratometry (K) obtained via optical biometry and topographic K collected from topography are two methods that have been applied recently 17, 18. However, whether these two K measurements yield similar refractive outcomes remains unknown. Because the different K measurements could cause refractive differences in specific IOLs 19, 20, the refractive outcomes of other K measurements may also differ to some extent, which needs further investigation.

As a consequence, the aim of the current study was to evaluate the refractive results between the topographic-derived K and biometric-derived K in a population that received EDOF IOL implantation. The preoperative risk factors for significant postoperative refractive error were also surveyed.

## Materials and methods

### Ethics declaration

The current study complied with the Declaration of Helsinki in 1964 and its consequent amendments. In addition, the current study was authorized by the Institutional Review Board of National Changhua University of Education (project code: NCUREC-112-052). The concern of written informed consent was postponed by the Institutional Review Board due to the retrospective design of the current study.

### Participant selection

A retrospective cohort study was conducted at the Nobel Eye Institute, which has more than 10 branches in Taiwan. The patients were enrolled if they fulfilled the following criteria: (1) age from 40 to 100 years, (2) had a diagnosis of complicated cataract or senile cataract, (3) underwent cataract surgery and EDOF IOL implantation at the Nobel Eye Institute, and (4) were followed up at the Nobel Eye Institute for at least one month. In addition, the following exclusion criteria were adopted to exclude patients with extreme conditions: (1) a preoperative best corrected visual acuity (BCVA) worse than hand motion, (2) the presence of central corneal opacity or corneal neovascularization, (3) the presence of end-stage glaucoma, (4) the presence of severe retinal disease, such as tractional retinal detachment or neovascular age-related macular degeneration, (5) the presence of optic disc atrophy and (6) the presence of amblyopia. The participants were subsequently divided into a biometric-K group and a topographic-K group according to the K measurement they received. Whether the patients received topographic K or biometric K measurements was based on the branch on which they received cataract surgery (some branches did not own the device for specific biometric K measurements). In addition, only the first eye that received cataract surgery was included in the current study. Finally, a total of 100 eyes from 100 patients were included in the current study, and 30 and 70 eyes were categorized into the topographic-K and biometric-K groups, respectively.

### Surgical details

All the cataract surgeries in the current study were completed by one experienced cataract specialist (C.-Y.L.), and two phacoemulsification devices were applied for cataract surgery. The IOL power was calculated via the Barrett formula because of the high accuracy reported in a previous study 21. The main incision was made via the superior approach method, and the ophthalmic viscoelastic device was then injected. After completing the continuous curvilinear capsulorhexis, hydrodissection and a side port incision were performed. The phaco-chop technique was used to remove the nucleus, and the residual cortex was then cleaned with an infusion‒aspiration probe. One type of EDOF IOL (TECNIS Eyhance, Johnson & Johnson, New Brunswick, New Jersey, United States) with or without toric function was implanted, and the residual ophthalmic viscoelastic device was removed. The hydroseal technique without suturing was utilized to close the wound, and the tobradex ointment was instilled at the end of cataract surgery. Postoperatively, prednisolone eyedrops, levofloxacin eyedrops and tobradex ointments were instilled for one week and then switched to combined dexamethasone and neomycin eyedrops for another week. Then, sulfamethoxazole and fluorometholone were applied for another three weeks.

### Ocular examination

All the patients who received cataract surgery underwent identical ocular examinations in any branch of the Nobel Eye Institute. The preoperative examinations included manifest refraction with UCVA and BCVA, cyclopegic refraction of both sphere and cylinder power by an autorefractor (KR-8900, Topcon, Itabashi-ku, Tokyo, Japan), intraocular pressure (IOP) by pneumatic tonometry (NT-530, Nidek Co. Ltd., Gamagori, Japan), central corneal thickness (CCT) of apex and thinnest parts, steep K, flat K, corneal cylinder power, angle kappa, scopic pupil diameter, corneal eccentricity index (CEI) and higher-order aberrations by a topographic machine (TMS-5, Tomey Corporation, Nishi-Ku, Nagoya, Japan), axial length (AXL), anterior chamber depth (ACD), lens thickness (LT), corneal diameter (CD) and “total K” (biometric K in this article) by a biometry machine (IOL Master 700, Carl Zeiss, Göschwitzer Str., Jena, Germany), endothelial cell density (ECD), and coefficient of variation (CV) and hexagonality (HEX) by a specular microscope (CEM-530, Nidek Co. Ltd., Gamagori, Japan). The postoperative examinations included UCVA at distance and near, BCVA if warranted, IOP, manifest sphere power and cylinder power, CCT, and the K value. The postoperative exams were also completed via devices identical to those used for the preoperative exams. The data before surgery, one week after surgery, and one month after surgery were collected. Notably, the toric IOL calculator version 2.0 provided by ASCRS was applied for all the toric IOL calculations in all branches of the Nobel Eye Institute. The spherical equivalent (SE) in the current study was set as the sphere power plus half of the cylinder power. In addition, visual symptoms, including halo, glare, starburst and ghosting after cataract surgery, were recorded.

### Statistical analysis

SPSS version 20.0 (SPSS Inc., Chicago, Illinois, USA) was used for the statistical analysis in the current study. The Shapiro-Wilk test was used to confirm the normality of the study population, which revealed a normal distribution (P > 0.05). The statistical power of the current study was 0.85, with a 0.05 alpha value and a medium effect size, which was assembled via G∗power version 3.1.9.2 (Heinrich Heine Universität at Düsseldorf, Germany). A descriptive analysis was performed to evaluate age, sex, preexisting ocular disorders, UCVA, BCVA, refraction status, topographic parameters, endothelial parameters and biometric parameters, and an independent t test was subsequently performed to compare these parameters between the biometric-K and topographic-K groups. Independent t tests were used to compare the postoperative visual acuity and refractive status between the two groups. The generalized linear model was used to evaluate the visual acuity and refractive status after cataract surgery between the two groups, and the adjusted odds ratio (aOR) with 95% confidence interval (CI) between the two groups was determined with adjustments for age, sex, topographic parameters, endothelial parameters and biometric parameters. In addition, the generalized linear model was also used to evaluate the correlation between preoperative factors and greater postoperative astigmatism in the two groups after adjustment for age and sex. Finally, the chi-square test was used to compare the ratio of dysphoptosia symptoms between the two groups. A P value < 0.05 was considered statistically significant and the P value lower than 0.001 was demonstrated as P < 0.001.

## Results

The baseline characteristics of the two groups are presented in Table 1. The mean ages were 64.49±10.63 years and 58.73±10.62 years in the biometric-K and topographic-K groups, respectively, which were not significantly different (P = 0.072). Additionally, the sex distribution and the ratio of systemic diseases did not significantly differ between the two groups (both P > 0.05). With respect to the other parameters, all the refractive, topographic, endothelial and biometric parameters presented similar distributions between the two groups (all P > 0.05) (Table 1).

One day postoperatively, the UDVA was 0.17 in the biometric-K group and 0.08 in the topographic-K group, which was a significant difference (P = 0.007). The postoperative SE on the same day was -0.18 in the biometric-K group and -0.28 in the topographic-K group, which were similar values (P = 0.537). Similarly, the postoperative cylinder power on the same day was not significantly different; it was -0.26 in the biometric-K group and -0.33 in the topographic-K group (P = 0.194). One month after cataract surgery, the UDVA was 0.15 and 0.07 in the biometric-K group and topographic-K group, respectively, which was still a significant difference (P = 0.040) (Table 2). Furthermore, the final SE was significantly greater in the biometric group (-0.42 versus -0.13, P = 0.019), and the final cylinder power was significantly lower in the topographic-K group than in the biometric-K group (-0.35 versus -0.13, P = 0.018) (Table 2). The multivariable analysis adjusted for the effects of age, sex and preoperative parameters revealed that the topographic-K group presented a significantly better UDVA (aOR: 0.819, 95% CI: 0.624-0.968, P = 0.044) and significantly lower cylinder power (aOR: 0.884, 95% CI: 0.761-0.949, P = 0.031) compared with the biometric-K group (Table 3).

With respect to the risk factors for postoperative astigmatism, angle kappa was significantly correlated with high postoperative astigmatism in the topographic-K group (P = 0.033) (Table 4). However, the angle kappa, steep K, and corneal cylinder powers were significantly correlated with high postoperative astigmatism in the biometric-K group (all P < 0.05) (Table 4). Five and two eyes experienced dysphotopsia symptoms in the biometric-K group and the topographic-K group, respectively, and the distributions of all dysphotopsia symptoms between the groups were not significantly different (all P > 0.05) (Table 5).

## Discussion

In brief, the results of the present study revealed similar postoperative SEs between the topographic-K group and the biometric-K group. However, the postoperative UDVA and residual astigmatism were significantly greater in the topographic-K group than in the biometric-K group. In addition, the angle kappa, steep K, and corneal cylinder powers were correlated with greater postoperative SE in the biometric-K group, whereas only the angle kappa was related to greater postoperative SE in the topographic-K group.

Postoperative residual astigmatism was significantly greater in the biometric-K group than in the topographic-K group in the current study. In a previous study in which the IOL master 500 was used, the mean residual astigmatism was approximately -0.97 D 7. Another study reported a residual astigmatism of approximately -0.40 D with the assistance of an IOL master 700 21. However, comparisons between different K measurements for postoperative residual astigmatism have not been reported. To our knowledge, this may be preliminary experience in demonstrating the relatively high accuracy of topographic-based K selection compared with biometry device-based K selection. In addition, the preoperative astigmatism and other parameters did not significantly differ between the two groups; thus, the homogeneity of the two groups may be adequate. Moreover, all the cataract surgeries were completed by one ophthalmologist, and the sites and widths of the corneal incisions were similar, if not identical, across all the cataract surgeries. As a consequence, the amplitude of surgical -induced astigmatism does not vary among all patients. In a previous study, the use of different optical coherence tomography-based biometers contributed to significantly different AXL and ACD results 22. In addition, the mean difference and repeatability of the K value can also differ across different IOL calculating devices 23, and the sphere IOL powers and K values are significantly different among biometers with different measurement techniques 24. Thus, different K measurements may alter the results of refractive outcomes after cataract surgery. The topography measures the K value within the 2.25-4-mm zone of the central cornea 25, and only the K value 3 mm from the central cornea was applied in the topographic-based K selection of the current study. Because central corneal astigmatism is different from mid-peripheral corneal astigmatism and theoretically influences visual acuity and refractive error to the greatest degree 26, the application of this real corneal curvature may benefit postoperative refraction. With respect to the trend of postoperative astigmatism change, the change in astigmatism did not exceed -0.25 D in the two groups. Since the change in postoperative astigmatism could result from the dislocation of the IOL 27, the minimal change in postoperative astigmatism in the two groups may imply the fair stability of this EDOF IOL.

With respect to postoperative visual acuity and SE, the postoperative UDVA was significantly different between the two groups. According to previous studies, the postoperative UDVA can degrade even with a low amount of postoperative residual astigmatism 28, and a postoperative astigmatism greater than -0.5 D could affect postoperative visual acuity 29. Consequently, the UDVA results of the current study were similar to those of previous studies 28, 29. However, the overall refractive outcome after the different astigmatism measurements was statistically insignificant 20, which may illustrate that the postoperative refractive status was affected by the method of astigmatism correction to a lesser degree. Consequently, the results of the current study regarding SE may correspond to those of previous studies 20. However, the trend of postoperative UDVA was similar between the two groups. Because cataract surgery is performed by one surgeon, the postoperative recovery of vision may not differ to a large extent. The postoperative hyperopia shift can partially indicate the degree of corneal edema, as determined in a previous study 30. In the present study, the degree of myopia one day postoperatively was -0.05 D and -0.11 D in the biometric-K and topographic-K groups, respectively, which indicated a low degree of corneal edema after surgery. Additionally, the total change in postoperative residual myopia was within -0.50 D in both the biometric-K and topographic-K groups, which may be an acceptable value after cataract surgery.

With respect to the possible risk factors for high postoperative astigmatism, angle kappa was correlated with greater postoperative astigmatism in patients with topographic-based K selection. Angle kappa is defined as the angle between the visual axis and the pupillary axis, which can alter postoperative visual and refractive outcomes 31. In addition, large angle kappa has a negative influence on postoperative vision after multifocal IOL implantation 32. Nevertheless, reports on the correlation between large angle kappa and greater postoperative astigmatism in EDOF IOL implantation are rare. The mean angle kappa of the patients in the current study was near -0.20 D, which is numerically smaller than that reported in a previous publication 32. Accordingly, even a population with a normal angle kappa could influence postoperative residual astigmatism to a certain degree. However, angle kappa, steep K, and corneal cylinder powers are associated with greater postoperative residual astigmatism in patients who receive biometry-based K selection for toric IOL implantation. A high K value correlated with greater residual astigmatism in a previous study discussing refractive surgery 33. In addition, the corneal cylinder power and corneal curvature are related to the degree of postoperative residual astigmatism after cataract surgery 34. The results in the biometric-K group in the current study correlated with the findings of previous studies 33, 34. Interestingly, only angle kappa influenced postoperative residual astigmatism in the topographic-K group, whereas two additional corneal parameters affected postoperative residual astigmatism in the biometric-K group. These results may indicate the high accuracy of topographic-based K selection for IOL calculation since it has lower postoperative astigmatism and is affected by fewer preoperative parameters.

Regarding the general surgical outcomes in the current study, the mean postoperative UDVA in the whole study population was 0.12 LogMAR. In previous studies, the mean UDVA ranged from 0.07 to 0.10 LogMAR, and the results of the present study were compatible with those of previous studies 35, 36. With respect to postoperative refraction, the mean SE was -0.37 D in the whole population, and the mean SEs reported in previous publications ranged from approximately -0.1 D to -0.20 D 9, 37. The postoperative residual astigmatism was -0.16 D in the topographic-K group, which is similar to the postoperative astigmatism reported in a previous study 18. Additionally, the biometric-K group had a postoperative residual astigmatism of -0.35 D, which was slightly inferior to the degree of astigmatism reported in a previous study 18. Regarding visual quality, 7% of patients experienced visual symptoms in the current study, and no IOL exchange was warranted to resolve the dysphotopsia. In previous studies, the dysphotopsia rate immediately after cataract surgery was greater than 10% 38. The above evidence may confirm the surgical quality of EDOF IOL implantation at our institution.

There are several limitations of the current study. First, the retrospective design of the current study could have resulted in a reduced homogeneity of the study population, although none of the preoperative parameters were significantly different between the two groups. In addition, the total number of eyes was only 100 in the current study, which may have contributed to some statistical bias, although the statistical power of 0.85 was not extremely low. Moreover, the postoperative topographic parameters and the biometric indices were not recorded in the current study because of its retrospective nature. Corneal astigmatism may worsen after cataract surgery, which is related to the steepest meridian of surgery 39, and the ACD increases after cataract surgery 40. Unfortunately, the retrospective design of our study cannot verify the results of previous studies. Consequently, the changes in these parameters cannot be assessed. Finally, all the participants in the current study were Han Taiwanese, and the external validity of the current study could be reduced.

In conclusion, compared with biometry-based K selection, topographic-based K selection is associated with lower postoperative residual astigmatism and better UDVA. Furthermore, topographic-based K selection was influenced by fewer factors regarding postoperative residual astigmatism. Consequently, topographic-based K selection could be recommended for patients with prominent preoperative astigmatism who are scheduled for EDOF IOL implantation. Further large-scale studies to evaluate whether the topographic-based K selection method is suitable for those with irregular preoperative astigmatism are needed.

## Figures and Tables

**Table 1:** Baseline features of the study populations

Feature	Biometric-K group (N: 70)	Topographic-K group (N: 30)	P
Age (year, mean ± SD)	64.49±10.63	58.73±10.62	0.072
Sex (male: female)	30:40	10:20	0.754
Laterality (right: left)	36:34	16:14	0.902
Systemic disease			0.139
Hypertension	18	0	
Diabetes mellitus	14	10	
Heart disease	2	2	
Other	2	4	
Eye surgery			0.544
Retinal surgery	2	0	
Refractive surgery	4	4	
UDVA (LogMAR)	0.71±0.33	0.57±0.25	0.079
CDVA (LogMAR)	0.51±0.30	0.44±0.22	0.643
IOP	14.80±2.63	14.14±3.10	0.351
Cycloplegia refraction (D)			
Sphere	-2.61±5.57	-2.87±5.77	0.727
Cylinder	-1.96±1.34	-1.35±0.90	0.153
SE	-3.59±5.36	-3.55±5.86	0.964
Topography			
Steep K	44.04±2.49	44.36±2.36	0.352
Flat K	43.04±2.27	43.35±2.17	0.310
Cylinder power	1.00±0.66	1.01±0.66	0.992
CCT	533.37±31.88	548.73±26.32	0.162
Angle Kappa	0.16±0.08	0.18±0.09	0.465
Pupil diameter	3.57±0.53	3.57±0.73	0.687
CEI	0.45±0.30	0.33±0.35	0.130
HOA	0.27±0.21	0.23±0.12	0.890
SA	0.16±0.41	0.20±0.22	0.907
AXL	24.97±1.74	24.52±1.61	0.568
ACD	3.22±0.41	3.11±0.44	0.403
WTW	11.81±0.36	11.96±0.34	0.195
LT	4.56±0.36	4.52±0.48	0.645
Biometric cylinder power	1.16±0.75	1.03±0.70	0.568
ECD	2741.40±246.29	2789.93±357.97	0.223
CV	30.86±4.99	30.07±3.95	0.734
HEX	67.42±5.32	69.47±6.06	0.270
Femtosecond Laser	14	8	0.713
Toric IOL implantation	32	12	0.765

ACD: anterior chamber depth, AXL: axial length, CCT: central corneal thickness, CDVA: corrected distance visual acuity, CEI: corneal eccentricity index, CV: coefficient of variance, D: diopter, HEX: hexagonality, HOA: higher-order aberrations, IOL: intraocular lens, IOP: intraocular pressure, K: keratometry, LT: lens thickness, N: number, SA: spherical aberration, SD: standard deviation, SE: spherical equivalent, UDVA: uncorrected distance visual acuity, WTW: white-to-white

**Table 2:** Postoperative visual and refractive outcomes between the two groups

Outcome	Biometric-K group (N: 70)	Topographic-K group (N: 30)	P
UDVA (mean ± SD)			
1 day	0.17±0.20	0.08±0.13	0.007*
1 week	0.16±0.19	0.08±0.14	0.034*
2 weeks	0.15±0.18	0.10±0.19	0.147
1 month	0.15±0.18	0.07±0.14	0.040*
SE (mean ± SD)			
1 day	-0.18±0.90	-0.28±0.77	0.537
1 week	-0.39±0.88	-0.28±0.68	0.513
2 weeks	-0.46±0.81	-0.18±0.58	0.055
1 month	-0.42±0.81	-0.13±0.41	0.019*
Cylinder (mean ± SD)			
1 day	-0.26±0.49	-0.33±0.64	0.194
1 week	-0.34±0.53	-0.28±0.67	0.288
2 weeks	-0.33±0.41	-0.25±0.55	0.200
1 month	-0.35±0.39	-0.16±0.52	0.018*

N: number, SD: standard deviation, SE: spherical equivalent, UDVA: uncorrected distance visual acuity

**Table 3:** Differences in visual and refractive outcomes between the two groups according to the multivariate analysis

Outcome	Biometric-K group (N: 70)	Topographic-K group (N: 30)	P
UDVA			
Crude OR (95% CI)	Reference	0.762 (0.589-0.937)	0.018*
aOR (95% CI)	Reference	0.819 (0.624-0.968)	0.044*
SE			
Crude OR (95% CI)	Reference	0.945 (0.717-1.294)	0.428
aOR (95% CI)	Reference	0.972 (0.829-1.267)	0.643
Cylinder			
Crude OR (95% CI)	Reference	0.853 (0.699-0.925)	0.017*
aOR (95% CI)	Reference	0.884 (0.761-0.949)	0.031*

aOR: adjusted odds ratio, CI: confidence interval, N: number, SE: spherical equivalent, UDVA: uncorrected distance visual acuity

**Table 4:** Correlations of preoperative factors with postoperative astigmatism in the two groups

Outcome	aOR	95% CI	P
Biometric-K group			
Steep K	1.225	1.017-1.465	0.028*
Flat K	1.107	0.968-1.332	0.641
CCT	0.944	0.901-1.075	0.729
Angle Kappa	1.213	1.064-1.382	0.017*
AXL	1.246	0.854-1.829	0.591
ACD	0.937	0.800-1.145	0.823
WTW	0.993	0.925-1.284	0.711
Femtosecond laser	0.918	0.738-1.101	0.215
Toric IOL	0.902	0.814-1.086	0.100
Topographic-K group			
Steep K	1.130	0.877-1.381	0.466
Flat K	1.001	0.823-1.473	0.654
CCT	0.958	0.840-1.107	0.932
Angle Kappa	1.198	1.008-1.492	0.033*
AXL	1.045	0.915-1.268	0.870
ACD	0.949	0.786-1.185	0.769
WTW	0.998	0.976-1.107	0.966
Femtosecond laser	0.934	0.818-1.209	0.312
Toric IOL	0.873	0.697-1.037	0.095

ACD: anterior chamber depth, aOR: adjusted odds ratio, AXL: axial length, CCT: central corneal thickness, CI: confidence interval, K: keratometry, IOL: intraocular lens, WTW: white-to-white

**Table 5:** Postoperative dysphotopsia symptoms in the two groups

Outcome	Biometric-K group (N: 70)	Topographic-K group (N: 30)	P
Halo	2	1	0.661
Glare	1	1	0.512
Starburst	2	0	0.488
Flash	0	0	0.999
Shadow	0	0	0.999

K: keratometry, N: number

## Abbreviations

ACD: anterior chamber depth
aOR: adjusted odds ratio
AXL: axial length
CCT: central corneal thickness
CDVA: corrected distance visual acuity
CEI: corneal eccentricity index
CI: confidence interval
CV: coefficient of variance
D: diopter
HEX: hexagonality
EDOF: extended depth-of-focus
HOA: higher order aberrations
IOL: intraocular lens
IOP: intraocular pressure
K: keratometry
LT: lens thickness
N: number
SA: spherical aberration
SD: standard deviation
SE: spherical equivalent
UDVA: uncorrected distance visual acuity
WTW: white-to-white
